# Virtual Surgical Planning, 3D-Printing and Customized Bone Allograft for Acute Correction of Severe Genu Varum in Children

**DOI:** 10.3390/jpm12122051

**Published:** 2022-12-12

**Authors:** Giulia Alessandri, Leonardo Frizziero, Gian Maria Santi, Alfredo Liverani, Dante Dallari, Leonardo Vivarelli, Giovanni Luigi Di Gennaro, Diego Antonioli, Grazia Chiara Menozzi, Alessandro Depaoli, Gino Rocca, Giovanni Trisolino

**Affiliations:** 1 Department of Industrial Engineering, Alma Mater Studiorum University of Bologna, 40136 Bologna, Italy; 2 Unit of Pediatric Orthopedics and Traumatology, IRCCS Istituto Ortopedico Rizzoli, 40136 Bologna, Italy; 3 Reconstructive Orthopedic Surgery Innovative Techniques-Musculoskeletal Tissue Bank, IRCCS Istituto Ortopedico Rizzoli, 40136 Bologna, Italy

**Keywords:** VSP, 3D-printing, in-hospital, point-of-care, patient-specific instruments, cutting guide, structural allograft, spondyloepiphyseal dysplasia, pediatric, osteotomy

## Abstract

Complex deformities of lower limbs are frequent in children with genetic or metabolic skeletal disorders. Early correction is frequently required, but it is technically difficult and burdened by complications and recurrence. Herein, we described the case of a 7-year-old girl affected by severe bilateral genu varum due to spondyloepiphyseal dysplasia. The patient was treated by patient-specific osteotomies and customized structural wedge allograft using Virtual Surgical Planning (VSP) and 3D-printed patient-specific instrumentation (PSI). The entire process was performed through an in-hospital 3D-printing Point-of-Care (POC). VSP and 3D-printing applied to pediatric orthopedic surgery may allow personalization of corrective osteotomies and customization of structural allografts by using low-cost in-hospital POC. However, optimal and definitive alignment is rarely achieved in such severe deformities in growing skeleton through a single operation.

## 1. Introduction

Complex deformities of lower limbs are frequent in children with genetic or metabolic skeletal disorders [1]. In particular, severe pathologic genu varum has been reported in conditions such as Infantile Tibia Vara (ITV or Blount disease), rickets, achondroplasia and multiple epiphyseal or spondyloepiphyseal dysplasia. Early correction is needed, but it is technically difficult and frequently accompanied by complications and recurrence. The advent of virtual surgical planning (VSP) and 3D-printing has revolutionized the approach to the diagnosis and treatment of several diseases in all fields of surgery [2,3]. Bony surgery represents the main field of application of these technologies, having demonstrated shorter surgery time, less intraoperative blood loss, fewer intraoperative fluoroscopies and a higher rate of excellent outcomes compared with conventional surgery [4,5,6]. Currently, the main advancement of VSP and 3D-printing regards the possibility to customize and personalize bone implants and devices for reconstructive surgery, especially in the presence of severe bone loss and large bone defects [7,8,9]. Another application of VSP is the acute correction of bony deformities through osteotomies, especially when multi-segmental, multifocal and multiplanar corrections are required [6,10]. In both these conditions the use of structural allografts is often required [11,12,13] and shape-matching is the primary criterion for selection of an allograft [14]. The entire process of planning of the surgical correction and shape-matching of the allograft may be optimized through the use of VSP and 3D-printing, allowing extreme personalization of the surgery and potential saving of donor bone tissue [15,16,17,18].

Herein, we present the case of a severe ITV in a 7-year-old girl that was treated by acute correction through bifocal osteotomy using VSP and 3D-printed patient-specific instrumentation (PSI) and customized structural allografts. The entire process was performed through an in-hospital 3D-printing Point-of-Care (POC).

## 2. Materials and Methods

### 2.1. Case Presentation

**Figure 1:**
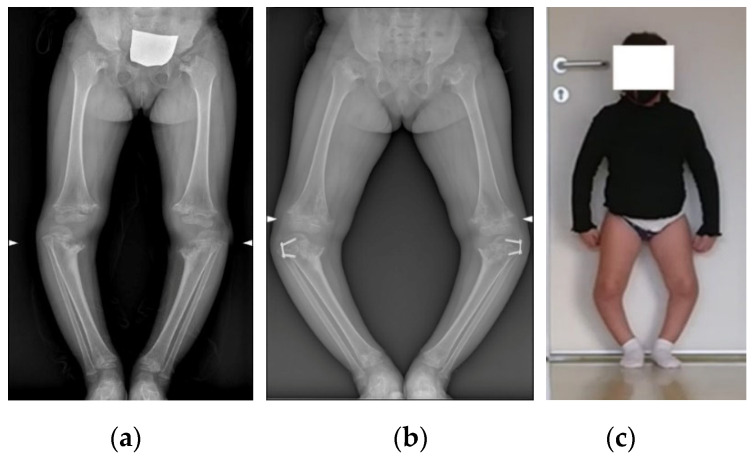
Preoperative longstanding radiographs of the patient (**a**) at the age of 4 years before tibial hemiepiphysiodesis with TBPs, (**b**) at the age of 7 years, and (**c**) clinical presentation at the age of 7 years.

A 4-year-old girl came to our observation in March 2019 for severe bowing of the knees in spondyloepiphyseal dysplasia (Figure 1a). She was treated by tibial hemiepiphysiodesis with tension band plates (TBPs), but two years and half later, in December 2021, the clinical condition was unchanged, and radiographs showed slight worsening of the deformity (Figure 1b,c). After discussion with the parents, acute correction with double osteotomy and structural allograft was indicated through VSP.

### 2.2. Image Acquisition and Reconstruction of 3D Model

**Figure 2:**
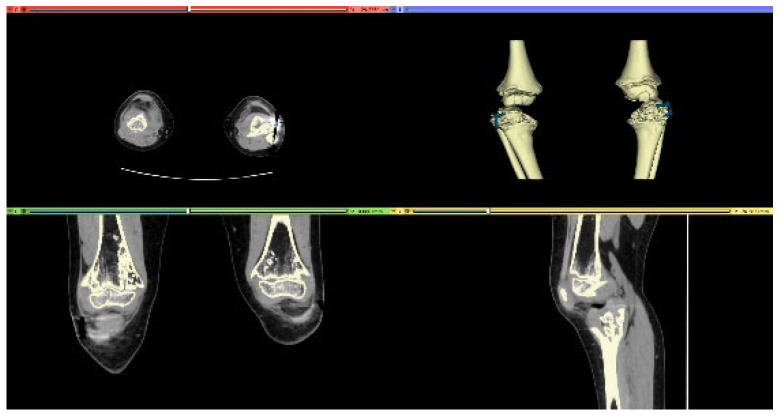
Segmentation on 3D slicer.

**Figure 3:**
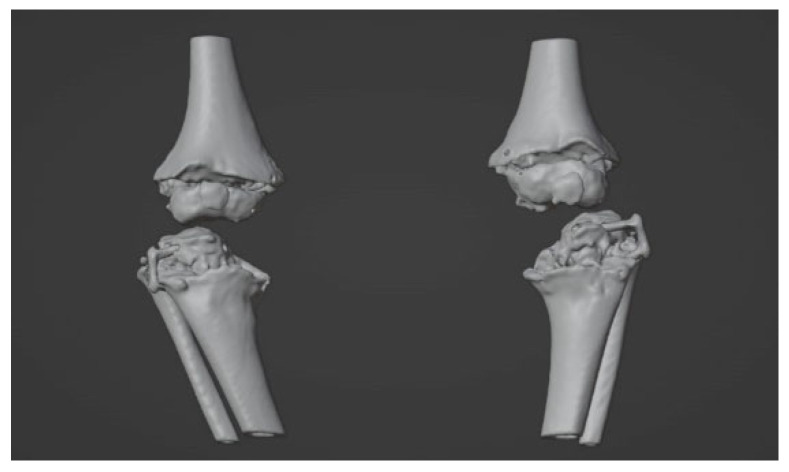
Model on Blender.

On long-leg weight-bearing anteroposterior and lateral radiographs, the initial malalignment of the knee and the degree of the correction were calculated. CT scans were obtained from both the knees by a low-dose protocol [19]. Acquisition was performed by obtaining scans with 5 mm slice thickness (0.625 mm reconstructions), that allows good accuracy for optimal reconstruction and modeling [20,21,22] while reducing the radiation dose (CTDI_vol_ = 13.3 mGy, DLP = 276.4 mGy∙cm). Medical images were acquired in the Digital Imaging and Communications in Medicine (DICOM) format and transferred to the 3D-printing POC. Three-dimensional digital reconstruction was achieved through a process of image segmentation and conversion in Standard Triangulation Language (STL) format, using open-source software (3D Slicer) [23], according to a well-established procedure in use at our lab [18,24,25] (Figure 2). In accordance with this procedure, the 3D digital reconstruction of the anatomical region of the patient is analyzed and imported in a virtual working environment (Blender) [26] (Figure 3) able to plan and simulate the correction, to design the patient-specific (PS) implants and/or device and to predict the final outcome.

### 2.3. Surgical Simulation and Planning

**Figure 4:**
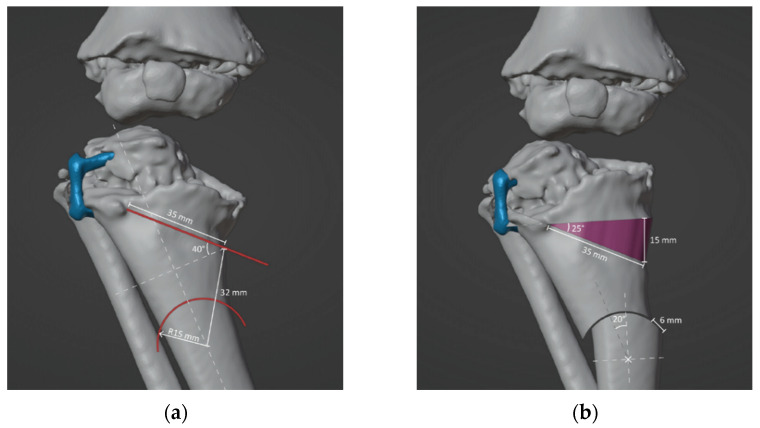
(**a**) Determination of position, direction and depth of the bone cuts. Proximal osteotomy was simulated by making a cut 18 mm from the inner end of the tibia, with an inclination of 40° to the horizontal axis of the tibia and a depth of 35 mm. The distal osteotomy was simulated by assuming a dome cut 32 mm below the starting point of the proximal osteotomy. The dome osteotomy was designed by imposing a radius of 15 mm to provide the widest contact area and lowest shift between the osteotomized bones. (**b**) Simulation of open wedge high tibial osteotomy and dome osteotomy.

Two orthopedic surgeons (G.T. and G.L.D.G.) and a design engineer (G.A.) defined the surgical strategy and desired correction. In brief, a double-elevating osteotomy according to a well-established surgical procedure was planned [27,28,29,30]. The first osteotomy was an elevation osteotomy of the medial plateau of the tibia stabilized with a wedge allograft, and the second osteotomy was a dome osteotomy below the level of the anterior tibial tuberosity. The size and position of the wedge, as well as the position and radius of curvature of the dome, were calculated in order to achieve an overall correction of the anatomic tibiofemoral angle (aTFA) of 45° (Figure 4).

**Figure 5:**
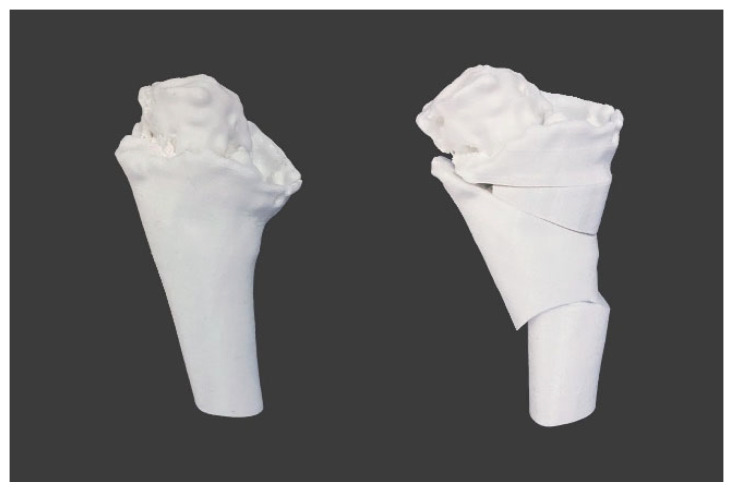
Three-dimensional printed models based on VSP before (**left**) and after (**right**) surgery.

The preoperative and postoperative models of the case were 3D printed (Figure 5).

### 2.4. Design and 3D-printing of the Patient-Specific Surgical Instrument

**Figure 6:**
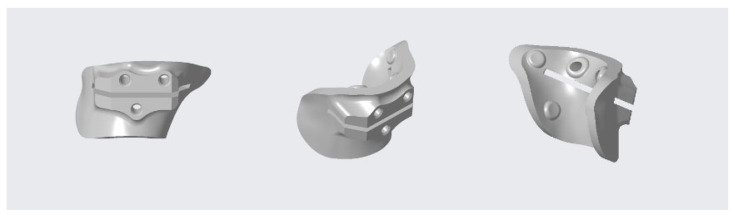
Patient-specific proximal osteotomy cutting guide.

**Figure 7:**
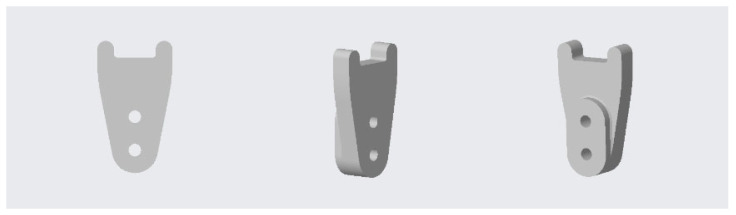
Patient-specific distal osteotomy cutting guide.

The basic infrastructure of our 3D-printing workstation consists of a low-cost 3D printer, a laptop and a digital workflow of opensource and commercial software for segmentation, modeling, planning, designing and 3D-printed PSI. Based on the planned correction, two patient-specific cutting guides were designed and produced in FiloAlfa^®^ PLA, according to the well-established method [31,32,33,34,35,36]. In brief, the first cutting guide was designed for the proximal osteotomy (Figure 6). The second cutting guide worked as a compass to perform a dome cut (Figure 7). Both these cutting guides were designed using the CAD environment (PTC Creo v8) and fabricated through FDM (Fused Deposition Modeling) 3D-printing technology.

**Figure 8:**
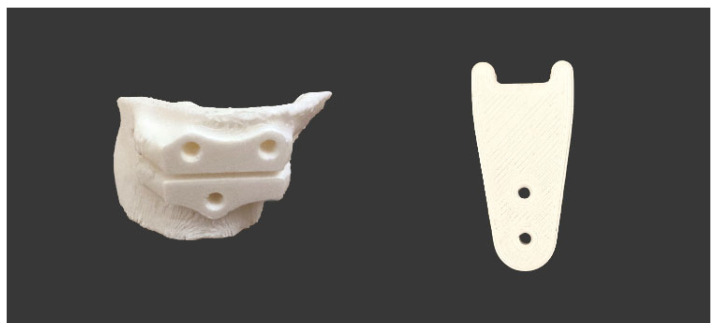
Three-dimensional printed cutting guides: for proximal osteotomy (**on the left**) and for distal osteotomy (**on the right**).

From the CAD software, a file in STL format was exported to be encoded by Ultimaker Cura v5.0.0, the slicing software. The models were loaded onto the FDM 3D printer (Delta Anycubic Predator). Using the parameters shown in Table 1, the objects shown in Figure 8 were obtained. Finally, the instruments were delivered to the sterilization center.

### 2.5. Design and Processing of the Patient-Specific Bone Allograft

**Figure 9:**
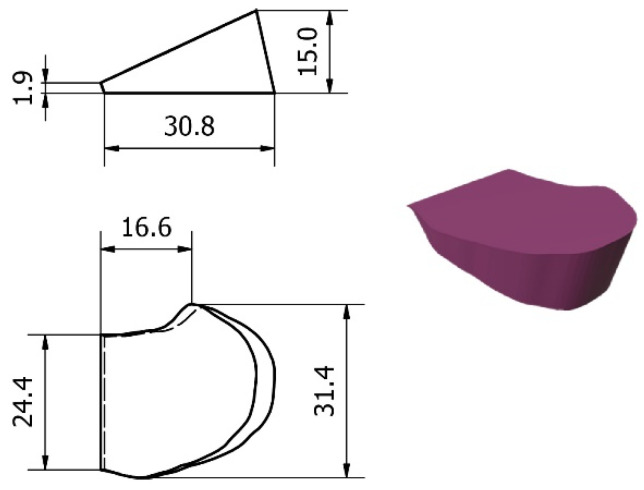
Bone wedge shapes and dimensions.

**Figure 10:**
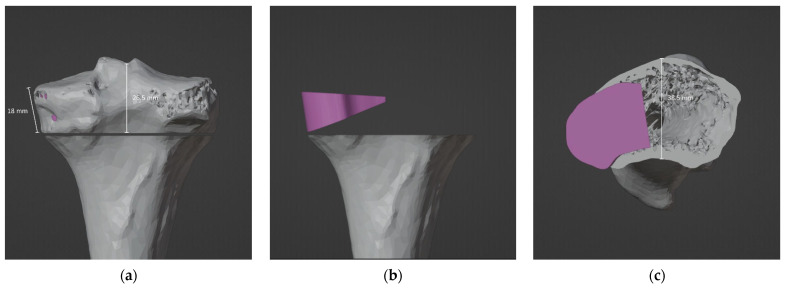
References for harvesting the bone wedge from the donor bone: (**a**) back view, (**b**) back view and (**c**) top view.

The VSP allowed to design the exact geometry of the allograft (Figure 9). In particular, the CT scans of the donor bones, recorded at the Musculoskeletal Tissue Bank (BTM) [37], were segmented and reconstructed in 3D in order to perform a manual selection of the bone segment that best matched the simulated wedge allograft (Figure 10).

**Figure 11:**
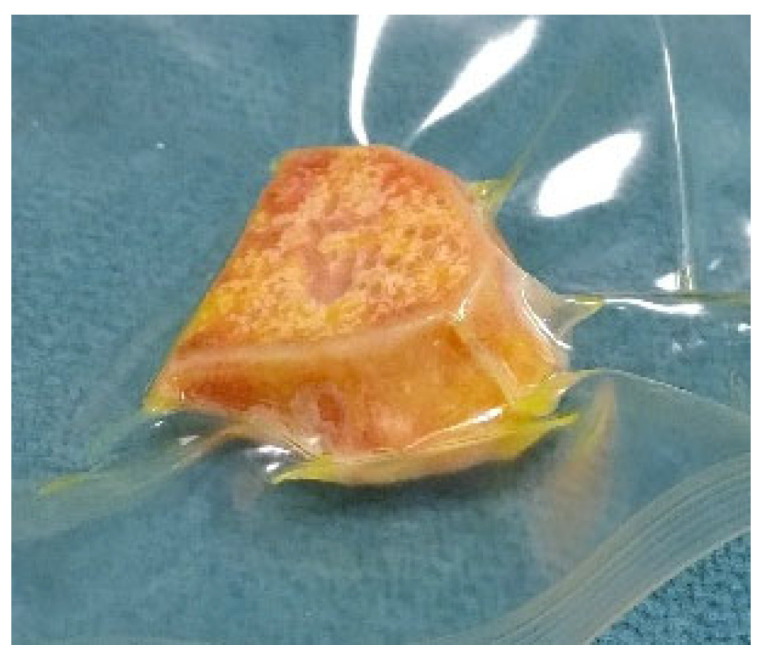
Custom bone allograft processed by BTM.

The wedge allograft was manually shaped in a cleanroom, according to the BMT standard procedures for processing, preservation, storage and distribution of a bone allograft. The allograft design, as well as aseptic processing, were performed in order to preserve the portion of cortical tissue, which allowed to obtain a load bearing suitable grafts to measure the size of the graft and to indicate the direction of implantation before packaging the graft sterilely (Figure 11).

## 3. Results

**Figure 12:**
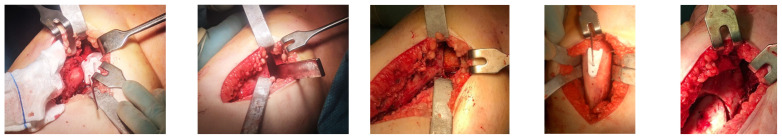
Step-by-step surgery procedure.

The patient had bilateral corrective surgery according to VSP, and the operation was replicated in the same manner in both knees. In brief, an initial shortening osteotomy of the fibula was performed in order to avoid overstress or dislocation of the tibiofibular joint. Then, a medial approach to the proximal tibia was used. The proximal elevation osteotomy and the dome osteotomy were performed using the cutting guides (Figure 12).

**Figure 13:**
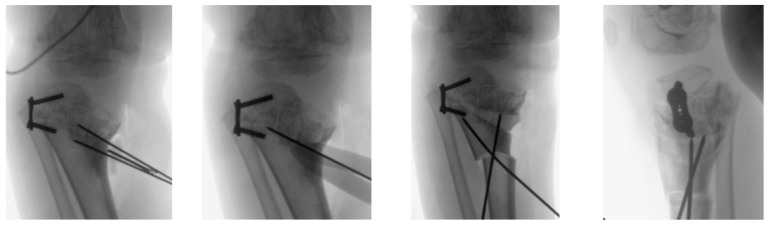
Final fluoroscopies.

**Figure 14:**
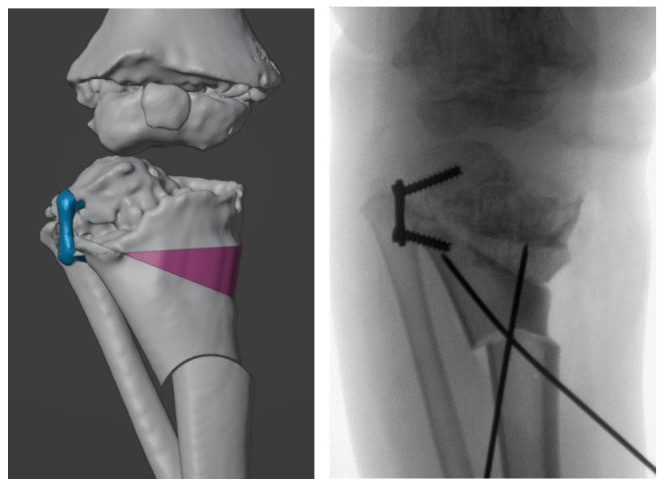
Comparison between the preoperative planning and the intraoperative fluoroscopic control.

**Figure 15:**
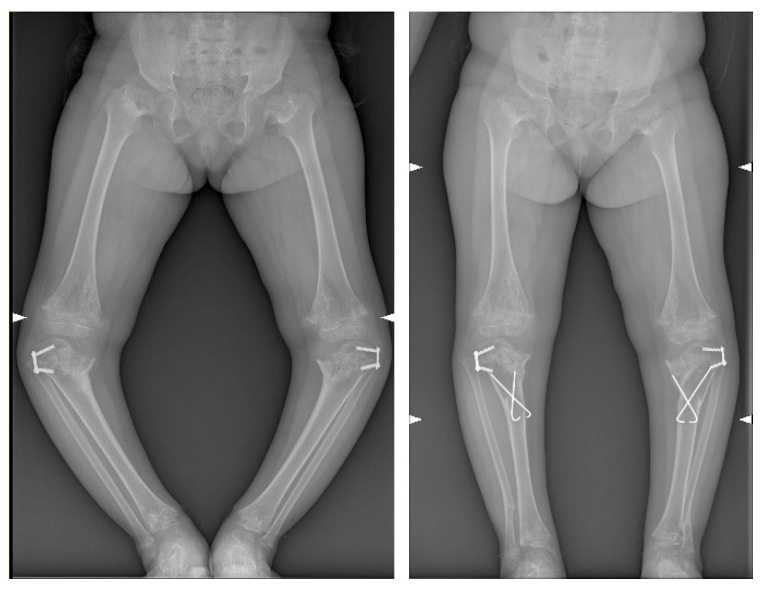
Preoperative and postoperative long-leg weight-bearing anteroposterior radiographs.

No significant deformation or mechanical problems of the cutting guides were observed. The wedge allograft was easily implanted without any intraoperative complication, and both the osteotomies were stabilized with two crossed 2.0 mm Kirchner’s wires. The lateral 8-plate was left in situ for further growth modulation (Figure 13). The intraoperative fluoroscopies showed that the final radiographic appearance closely resembled the preoperative planning (Figure 14). However, the postoperative long-leg weight-bearing radiographs showed incomplete correction (Table 2) (Figure 15).

## 4. Discussion

The present study describes an innovative step-by-step process for performing a PS double-tibial osteotomy in severe infantile genu varum by using VSP, 3D-printing and a customized structural allograft. We performed the entire process by using an in-hospital low-cost 3D-printing POC.

Currently, there is initial but converging evidence that VSP and 3D-printing result in highly accurate correction following femoral and tibial osteotomies in adults. In a recent systematic review, Aman et al. [6] reported that five comparative studies out of six demonstrated that, compared to conventional osteotomies, PS knee osteotomies had significantly more accurate correction, shorter operative times, decreased intraoperative fluoroscopy and low rates of complications. Additionally, Raza et al. [10] confirmed this trend in pediatric osteotomies.

On the other hand, the need for preoperative CT scans, the costs, safety and immediate availability of this technology still limit the use of VSP and 3D-printing in daily surgical practice. To overcome these issues, we used a very low-dose protocol that allows to obtain very accurate bone reconstruction and more informative 3D visualization. Furthermore, we developed a low-cost 3D-printing POC for rapid on-demand prototyping of bony models and PSI, directly available in the medical office. The development of in-house 3D-printing POC for producing patient-specific devices is rapidly growing, with promising results, suggesting that this technology has reached maturity and could set aside many commercially available systems. On the other hand, certification of the entire process of planning, simulating and 3D-Pprinting is required, for example, in the European community, thus limiting the use of open-source software and low-cost 3D printers for surgical applications [38]. Comparative studies and clinical trials should be promoted to demonstrate the superiority of commercial systems compared to freely-available resources. Concurrently, standardized and certified procedures and devices must be established in order to consolidate the role of 3D-printing POC in standard clinical practice as a viable alternative to outsourced professional solutions.

A key point of our VSP driven approach is the matching of the wedge allograft to the patient unique anatomy. This allowed us to achieve a precisely shaped graft with a tri-cortical outer plater to provide immediate structural support and a dense cancellous body. This peculiar aspect can provide increased stability to the construct, faster recovery, reduced graft subsidence and decreased risk of recurrence. Another advantage is that the use of a tricortical allograft, along with a dome osteotomy, can minimize the need of bulky hardware, especially in children with small bones and irregular ossification.

Incomplete evidence remains regarding the use of VSP for correcting severe knees deformities in children. In our case, we were able to accurately replicate the planned surgery at the time of the operation by using the PS cutting guide and customized bone grafts. Nevertheless, at the latest follow-up, one year later, the patient showed a slight recurrence of varus deformity, particularly on the left side. This could be due to many factors: (1) the specific features of the disease, (2) age, (3) inappropriate osteotomy type and (4) insufficient correction and/or lack of overcorrection. As observed in the treatment of ITV, there is a very high incidence of recurrence of varus deformity in children, and most authors recommend the correction to be made before age 4 [39]. Moreover, previous studies in ITV recommended an average overcorrection of 15° for preventing a relapse [40]. In such severe cases, the use of gradual correction by external fixator would be preferred, but the advantages and disadvantages of this technique, as well as the experience and preference of the surgeon, must be considered [41]. The methodology applied in this case report could be used to evaluate the impact of a precisely dosed and reproducible overcorrection when surgery must be performed between the ages of 4 and 12.

## 5. Conclusions

In conclusion, corrective osteotomies for infantile genu varum may be improved in terms of accuracy, personalization and safety by using VSP and 3D-printing technology. We demonstrated that our all-in-house 3D-printing PoC was effective and safe in producing the PS instrumentation and customized allograft. The implementation of 3D-printing PoC may also increase the use of 3D-printing technology in pediatric orthopedic surgery, resulting in cost and time savings, but common regulatory standards must be ensured for ensuring the efficacy, efficiency and safety of the entire process.

## Figures and Tables

**Table 1:** Parameters set for 3D-printing. PLA: Polylactic Acid.

Parameters	Value
Printing (Nozzle) Temperature	200 °C
Heated Bed Temperature	60 °C
Nozzle Diameter	0.4 mm
Layer Thickness	0.2 mm
Printing Speed	60 mm/s
Infill Density	100 (%)
Flow	100 (%)
Support	Yes
Material	FiloAlfa^®^ PLA

**Table 2:** Details of the preoperative and postoperative radiographic measurements (Figure 15). MAD: mechanical axis distance; aTFA: anatomical tibiofemoral angle; FC-TS angle: femoral condyle-tibial shaft angle.

	MAD		aTFA		FC-TS Angle	
	preop	postop	preop	postop	preop	postop
right	−100 mm	−39 mm	−45°	−3°	49°	86°
left	−100 mm	−51 mm	−44°	−15°	55°	72°

## Data Availability

The datasets generated during the current study are available from the corresponding author upon reasonable request.
